# Mobile Technology for Community Health in Ghana: what happens when technical functionality threatens the effectiveness of digital health programs?

**DOI:** 10.1186/s12911-017-0421-9

**Published:** 2017-03-14

**Authors:** Amnesty E. LeFevre, Diwakar Mohan, David Hutchful, Larissa Jennings, Garrett Mehl, Alain Labrique, Karen Romano, Anitha Moorthy

**Affiliations:** 1 Department of International Health, Johns Hopkins School of Public Health, 615 N. Wolfe Street, Baltimore, MD 21205 USA; 2 Department of International Health, Johns Hopkins University Global mHealth Initiative, 615 N. Wolfe Street, Baltimore, MD USA; 3 Grameen Foundation Ghana, OSDTD5041 No. 25 Labone Cresent, Accra, Ghana; 4 World Health Organization, Geneva, Switzerland

**Keywords:** Digital health, mHealth, Maternal and child health, Frontline health workers, IVR messaging

## Abstract

**Background:**

Despite the growing use of technology in the health sector, little evidence is available on the technological performance of mobile health programs nor on the willingness of target users to utilize these technologies as intended (behavioral performance). In this case study of the Mobile Technology for Health (MOTECH) program in Ghana, we assess the platform’s effectiveness in delivering messages, along with user response across sites in five districts from 2011 to 2014.

**Methods:**

MOTECH is comprised of “Client Data Application" (CDA) which allows providers to digitize and track service delivery information for women and infants and “Mobile Midwife” (MM) which sends automated educational voice messages to the mobile phones of pregnant and postpartum women. Using a naturalist study design, we draw upon system generated data to evaluate message delivery, client engagement, and provider responsiveness to MOTECH over time and by level of facility.

**Results:**

A total of 7,370 women were enrolled in MM during pregnancy and 14,867 women were enrolled postpa1rtum. While providers were able to register and upload patient-level health information using CDA, the majority of these uploads occurred in Community-based facilities versus Health Centers. For MM, 25% or less of expected messages were received by pregnant women, despite the majority (>77%) owning a private mobile phone. While over 80% of messages received by pregnant women were listened to, postpartum rates of listening declined over time. Only 25% of pregnant women received and listened to at least 1 first trimester message. By 6–12 months postpartum, less than 6% of enrolled women were exposed to at least one message.

**Conclusions:**

Caution should be exercised in assuming that digital health programs perform as intended. Evaluations should measure the technological, behavioral, health systems, and/or community factors which may lead to breaks in the impact pathway and influence findings on effectiveness. The MOTECH platform’s technological limitations in ‘pushing’ out voice messages highlights the need for more timely use of data to mitigate delivery challenges and improve exposure to health information. Alternative message delivery channels (USSD or SMS) could improve the platform’s ability to deliver messages but may not be appropriate for illiterate users.

**Trial registration:**

Not applicable.

**Electronic supplementary material:**

The online version of this article (doi:10.1186/s12911-017-0421-9) contains supplementary material, which is available to authorized users.

## Background

Mobile phones are the leading form of communication worldwide [1]. Their widespread and increasing use, particularly in low and middle income countries where the disease burden is highest, has led to growing calls to harness the potential of mobile and wireless technology to improve health and health care delivery. Mobile-health (mHealth)—defined as the use of mobile and wireless technology for health [2]—aims to improve health outcomes by addressing critical health systems constraints to service delivery, coverage, and utilization [3].

Throughout the last decade, over 600 mHealth pilot strategies and programs have been introduced globally [4]. Despite the proliferation of mHealth programs, evidence on their effectiveness is limited [5–7] and only modest gains have been observed for interventions aiming to improve provider diagnosis and management, and/or increased user demand through messaging using automated voice or SMS [7]. While calls to address broader evidence gaps in linking digital technologies to outcome and impact level health indicators are emerging, little to no attention has been paid to improving program monitoring, including measuring critical processes on the technological and behavioral performance of the program.

The technological performance aims to determine if the technology platforms performs as intended, whether in aiding program implementation or generating data critical to monitoring and evaluation. Depending on program design, technology platforms may aim to deliver health information messages, alerts and reminders, and/or support the collection of data, including patient-level information on the timing and content of services provided. Variability in how the technology platforms are designed and maintained, including decisions on which data elements are collected, with what frequency, by whom, and for what purpose, greatly influences the consistent delivery of messages and routine monitoring of programs.

The behavioral performance of a program is characterised by the interaction of users, including health care providers and patients, with the technology and program delivery strategy in a dynamic and evolving health systems and community context. For mobile technologies aiming to improve supply side dimensions of service delivery, providers must be willing and able to utilize the technology; a factor which is influenced by perceptions on usability, feasibility and acceptability within a given health systems context [8]. For mHealth solutions which aim to improve demand for and utilization of services through messaging apps, users must not only receive messages (technological performance) which may require negotiating use of the phone if shared (community/household factors) but then decide to open, listen to and/or read them once received (behavioral performance). Whether the technology corresponds to a change in health behaviour and practices requires a complex negotiation of social norms/practices, household and community dynamics, as well as overcoming financial and physical barriers to care.

Collective consideration of the technological and behavioral performance of an mHealth program is essential for accurately measuring program effectiveness and protecting against ‘program drift’—defined as the deviation from intended design in real-world delivery of programs resulting in decreased benefit for patients [9]. In the absence of digital technologies, including mobile phones, data derived from (paper-based) special surveys or health information systems may be used to measure effectiveness, including coverage, or inform process evaluations aimed at assessing fidelity and implementation quality, amongst other factors [10]. The use of mobile and other digital health strategies have the potential to generate data in real-time on essential processes as well as patient careseeking and health status, enhancing not only program implementation and monitoring but evidence needed for summative evaluations.

**Fig. 1:**
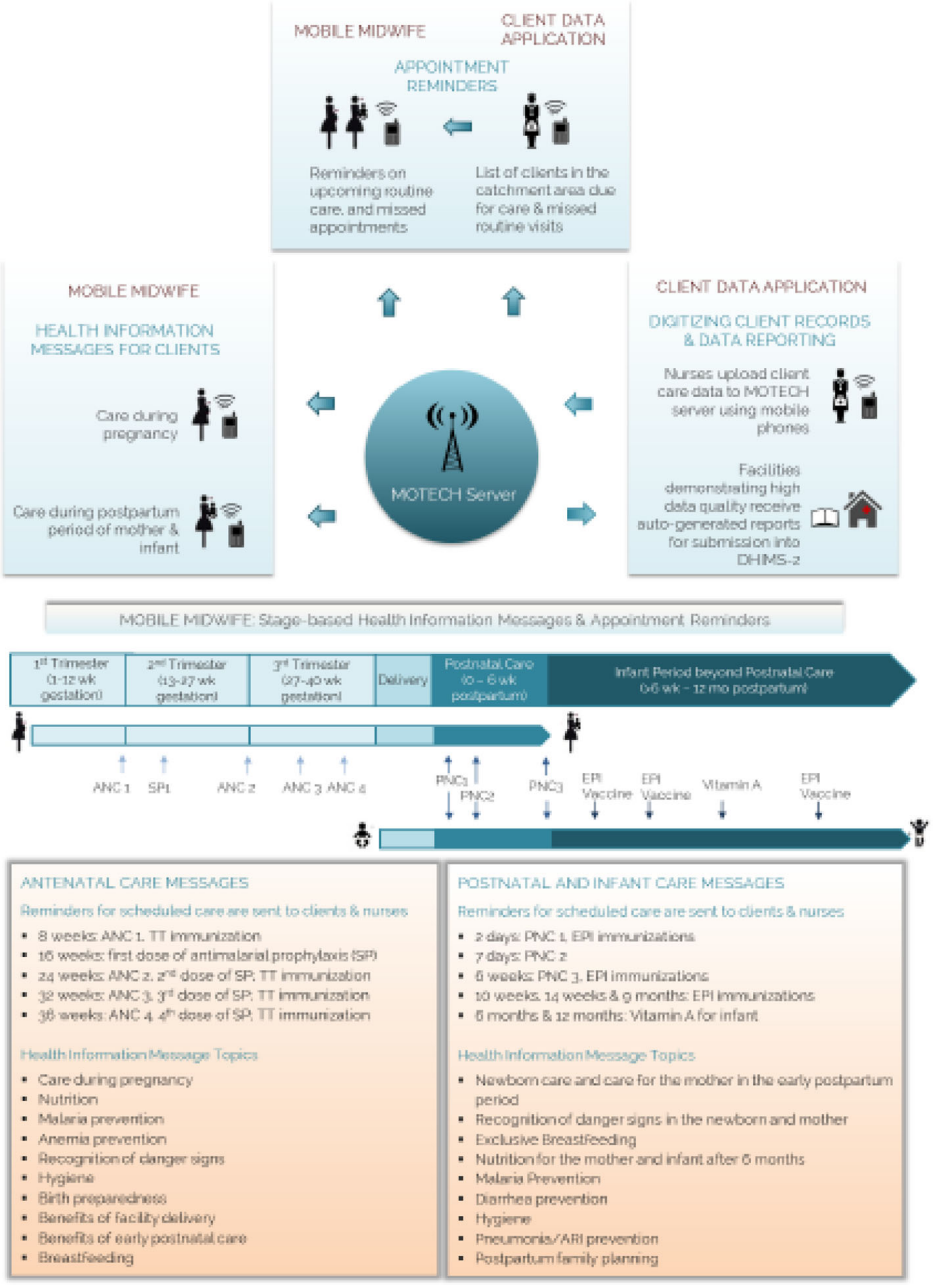
Mobile Technology for Health in Ghana: program overview

The Mobile Technology for Community Health (MOTECH) program was initiated in 2009 in collaboration with the Grameen Foundation and the Ghana Health Services (GHS) to utilize mobile technology to improve uptake and quality of care of maternal, newborn and child health services (MNCH). The MOTECH platform consists of two interconnected mobile applications– “Mobile Midwife” and the “Client Data Application" (hereafter referred to as Client Data App) (Fig. 1). Mobile Midwife enables pregnant women and mothers of children under age one to receive pre-recorded audio messages on MNCH education and care in local languages or SMS messages on their mobile phones timed to a women's gestational age or that of her infant's. The Client Data App enables frontline health workers to use mobile phones to ﻿identify women and infants in their area that are due or overdue for care ﻿﻿and ﻿﻿improve data reporting processes ﻿by digitally recording care given to patients. In this case study, we draw from system generated data collected across five districts in Ghana from 2009 to 2014 to explore two research questions: (1) does the MOTECH platform perform as intended in delivering audio messages to registered users across study sites and over time? and (2) how do rates of active listening among users differ across study sites, during pregnancy and postpartum, and across thematic content areas? By exploring these research questions on the technological and behavioral performance of MOTECH respectively, we hope to inform efforts to optimize the use of technology for health in low resource settings where disease burden is highest.

## Methods

### Study context and population

Ghana is home to a population of almost 25 million disbursed across 216 districts in 10 administrative regions [11]. Over 40% of Ghanaians are under the age of 15 and over half live in urban areas [11]. While child mortality rates have declined by 50% since 1990, for every 1,000 live births 62 children die; 47% within the first 28 days of life [12]. Similar declines in maternal mortality have been observed over the last three decades, however, the lifetime risk of maternal death remains high at 1 in 66, and for every 100,000 live births an estimated 380 women die [12]. Observed declines in mortality have been driven by concurrent increases in the utilization of critical MNCH services, yet gaps in continuity of care during pregnancy, delivery, and postpartum persist. Despite near universal attendance of at least 1 antenatal care (ANC) session and high utilization of 4 or more ANC visits (84%), nearly 30% of deliveries are not attended by a skilled birth attendant, and nearly 20% do not receive postnatal care (PNC) [12].

Field level implementation of the MOTECH program launched in August of 2010 in Kassena-Nankana West (KNW) district in the Upper Each Region of Ghana, and replicated in 2011 in Awutu Senya District in the Central Region. In 2012, with added funding from USAID and the Bill and Melinda Gates Foundation (BMGF), MOTECH was expanded into new districts: Gomoa West in Central region, Dangme East in Greater Accra region, South Tongu in the Volta region. Administrative division of two districts occurred during implementation; effectively splitting Awutu Senya into Awutu Senya East and West and Dangme East into Ada East and West and raising the total number of program districts to seven across four regions.

### Program overview

Figure 1 provides an overview of the MOTECH program and its two inter-related components: Client Data App (supply side) and Mobile Midwife (demand side).

The Client Data App consists of simplified digital and paper registers consolidating information previously collected in over a dozen paper based registers in Health Centers and Community-based Health Planning and Services (CHPS) facilities. The Client Data App is a supply side intervention which allows facility and community based providers to record all care provided into five simplified paper registers, and digitize clinical care information pertaining to MNCH and other essential care to better track and deliver care to women and infants. Frontline health workers record patient care data onto mobile devices and upload these data to the server (Additional file 1: Figure S1). Data collected by health workers using Client Data App are uploaded into MOTECH’s central database and cross-checked against GHS guidelines on routine care needs for pregnant women, infants, and lactating mothers to trigger a system of alerts about upcoming and missed care sent to both clients in Mobile Midwife program and health workers [13]. These alerts are sent as weekly short message service (SMS) lists containing Mobile Midwife clients with their IDs and care they require. Field teams, composed of Grameen Foundation and district health data staff, routinely monitored and scored health facilities on the accuracy and completion of clinical information uploaded into the MOTECH server compared to care captured in the paper-based registers. Facilities in MOTECH districts that attained 85% completion and accuracy ratings for reporting for three consecutive months were eligible to be ‘automated.’ Automated facilities received auto-generated monthly reports containing aggregate care. This threshold for automation was determined in collaboration with GHS, and based on government standards for data quality.

Mobile Midwife is a demand side component of the MOTECH program which aims to improve client knowledge and awareness of key health information during pregnancy and postpartum period, with the goal of stimulating best practices and encouraging timely and appropriate service utilization. Mobile Midwife is comprised of a maximum of 88 stage-based educational messages and care alerts for pregnant and postpartum women timed to their gestational age or age of their infants, respectively. Educational content was developed based on global and national MNCH guidelines and varied slightly between districts in order to debunk local cultural beliefs and practices. Clients received messages as automated voice recordings in local languages or SMS messages at a day and time of their choosing every week. The voice messaging service was persistent. A weekly message was resent to a registered phone number if a client listened to the call for less than 30 s, either because they missed the call, the device was powered off, there were call congestion issues during message delivery, or if the mobile network was temporarily down. In this case, the system was designed to send that message multiple times over the subsequent two hours, and once every other day until the next message is due.

Mobile Midwife and Client Data App are underpinned by a larger technology infrastructure which includes platform support and server hosting. The MOTECH platform is based on a modular, extensible open-source software comprised of a core platform and several modules, which were developed in 2009 and upgraded in 2012. Specific details on system architecture and evolution over time are available elsewhere, including security aspects and available modules [13, 14]. The software development responsibilities were split between two teams: the server-side components were designed by a group from the University of Southern Maine, and the mobile phone components were designed by a young company based in Ghana [13]. Following the initial development of the overall system architecture, the platform and server hosting were maintained at the Grameen Foundation offices in Accra, Ghana.

### Program start-up and implementation

The MOTECH program was developed and implemented in collaboration with GHS, regional and District Health and Management Teams (DHMT). Table 1 summarizes program activities and inputs required to develop, startup and sustain implementation of the MOTECH program, including Mobile Midwife and Client Data App. To initiate project activities at a district level, extensive profiling was first undertaken to gather data on the health systems and telecommunications infrastructure. Content initially developed at a national level was localized to specific regional deployments and mobile phones^1^ were procured (Nokia 1680c-2, Nokia 2330c-2, Nokia C1-01, Nokia Asha 200, Nokia 2330c-2 s) for facility-based providers. Orientation of district-level leadership and facility-based provider training was undertaken. As part of Mobile Midwife, community health volunteers and facility level providers^2^ were trained to register clients to receive stage-based actionable advice as well as service delivery alerts and reminders. Training in the use of Client Data App included DHMT, regional, and national level staff as well as facility-based providers in 69 CHPS facilities and 23 Health Centers/Hospitals. Trainings were followed by a three-month practice interval which allowed providers time to practice, adjust to the five simplified paper registers, and acclimate to data entry and uploading on mobile phones. To facilitate the trouble-shooting of technical and/or programmatic issues, a customer support service was established and maintained at the Grameen Foundation offices in Accra, Ghana. This service provided support for health workers using Client Data App, and tracked overall data uploads, and flagged changes in data upload trends.^3^ Once facility-based providers demonstrated proficiency in using MOTECH’s Client Data App, Mobile Midwife activities were initiated. At a community level, district launch events inclusive of key political figures, religious leaders, health workers and community members were held through durbars (festivals) and other marketing events to promote the program and encourage registration. Posters and murals were additionally installed on the exterior walls of community structures to raise awareness and promote registration into Mobile Midwife.

**Table 1:** Description of MOTECH program activities and inputs

Program Activities	Description and Inputs
Development	
Program Design	National leadership meetings held among central program leadership, regional and district health management teams
Content development, provider training materials	GF staff worked with GHS Family Health Division and Health Promotion Unit at National level, and with District counterparts to develop messaging content, training materials, and marketing materials
Telecommunications	• Voice message program national negotiations and ongoing partnership with Telecommunications Companies • Infrastructure establishment to manage call system
Technology	Platform support and server hosting
Personnel	Central, Regional, and District staff time allocated to develop the program for each district
Start Up	
National level	Established MOTECH National Steering Committee
District Profiling	Data on health system and telecommunication infrastructure compiled by DHMT for central database
District capacity building	Establish partnerships with Regional and District Health Management Teams • Establish Regional MOTECH sub-Steering Committee for Greater Accra and Volta regions • Establish district Technical Working groups • Placement of 1 full time GF staff in each district and concurrent appointment of one DHMT staff member to serve as “District MOTECH Coordinator” and lead day to day operations. Both staff work under direction of District Director of Health.
Content Localization	Standardization, translation, and testing of Voice health messaging content
Equipment	Phone purchases for facilities
Customer Support	Customer service referral system for technical or programmatic issues
Training	• Orientation for leadership • Nurse training on Simplified Registers and technical data entry application, • Sub-District orientation on program
Community Mobilization	District Launch events, Durbars and other marketing
Partnership Building	• Regional steering committee meeting for program planning in district; • Technical steering committees in the district meet monthly for 4 months to direct start up
Vehicle Maintenance	Cost to maintain and use existing vehicles without new capital cost purchase
Office Maintenance	Central Office space
Telecommunications	Airtime for voice messages, Nurse data upload cost
Personnel & Benefits	Grameen, Regional, and District staff time allocated to initiating the program for each district
Technology	Platform support, server hosting, system modification to absorb call capacity
Implementation	
Technical Groups	DHMT included program tasks in current workflow
M&E	Routine data entry application for use in monitoring
Continued Training	Refresher training, Training of new hires
Equipment & Materials	Replacement/resupply of phones and Simplified Registers
Field Office Maintenance	GHS District Office space
Office Maintenance	Central Office space— Grameen Foundation
Personnel & Benefits	Central, Regional, and District staff time allocated to maintaining the program
Telecommunications	Airtime for voice messages, Nurse data upload cost
Technology Maintenance	Data platform, IT technical assistance

^*^Modified from Willcox M et al. 2017 (Willcox M, et al. Is Mobile Technology for Community Health good value for money? Evidence on the cost effectiveness of mobile health in Ghana. Submitted for publication)

Following start-up activities, health facilities began patient registration and enrollment into MOTECH. Providers were encouraged to enter and upload patient level information on a daily basis to facilitate the sending of alerts and reminders. In two districts (Awutu Senya East and Awutu Senya West), high performing facilities were automated and thus given the opportunity to receive monthly feedback reports from Grameen Foundation, summarizing service delivery statistics. To support facility-based activities routine district level meetings were held and supervision visits made to support facility level providers. Where possible, DHMT members sought to include program tasks in their current workflow. However, the Grameen Foundation provided additional program support including device replacement, refresher training, and routine data entry, management and feedback.

### Study design and measurement

**Fig. 2:**
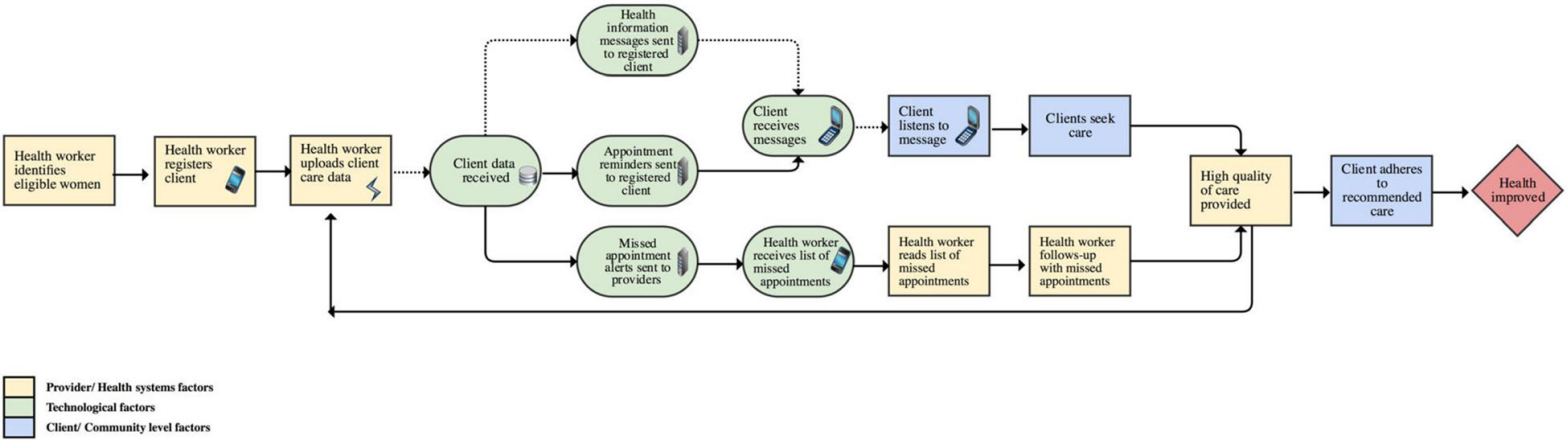
Measuring program fidelity: was the program delivered as it was intended?. The dotted line denotes the pathway assessed as part of this manuscript. Yellow boxes denote factors which are influenced by health systems and/or providers, the light green represents technological factors, and the light blue community/client level factors

Using a naturalistic study design, this case study sought to describe variation across study sites over time in the receipt of and engagement with health information messages [15]. Figure 2 presents our conceptual framework for assessing the optimal pathway between health care providers who identify and register clients; the technological platform that receives and sends health information messages as well as alerts and reminders for care; end-users who receive and access messages; service delivery and careseeking; and ultimately, improved health status. In practice, multiple breaks are likely to have occurred at each point along this pathway as a result of technological (network coverage, feasibility/usability of the device, size of the data bundle, phone functionality/access); behavioral (user’s willingness to listen to/access messages, motivation/satisfaction); health systems (workload, organizational and structural inputs); and/or community constraints (financial/physical barriers to care, social norms, power dynamics). In this analysis, we draw upon system generated data to explore the chain of events denoted by a dotted arrow linking provider registration, message delivery, and receipt.

Technological performance was assessed by exploring the MOTECH platform’s effectiveness in ‘pushing’ out messages across the continuum of care and by content area to registered women. Technological indicators included the proportion of messages ‘pushed out’ out of the total number of messages expected to be ‘pushed’ (Additional file 1: Figure S1 and Additional file 2: Table S1). Behavioral performance was assessed by capturing trends in provider and client engagement with Client Data App and MM, respectively. To measure provider engagement with Client Data App and ascertain the broader feasibility of recording patient-level health information, we assessed the frequency and volume of data uploads over time and by level of facility. To explore client engagement, we identified the proportion of and characteristics of message recipients who listened to at least 50% the length of each message received (defined as ‘active listeners’).

### Data collection extraction and analysis

Data from Mobile Midwife and Client Data App were obtained from MOTECH server housed at Grameen Foundation offices in Accra, Ghana. Data elements specific to Client Data App included health facility data uploads, while data from Mobile Midwife included data on health information messages, including messages sent and accessed by pregnant women and mothers of infants. Messaging access was defined by the number of clients who ‘listened’ to stage-based health information messages successfully ‘pushed out.’ Since sharing of phones and phone numbers is common practice in Ghana, many clients were listed under the same phone numbers. For the purposes of this analysis, we extracted details of ‘listening’ for unique phone numbers to facilitate client level analysis. We further excluded registered women who did not have their own or have access to a mobile phone in their household and insteadhad to rely on a community phone to call into the system to retrieve their weekly messages. As part of the Client Data App, facility based providers are required to upload data on the careseeking practices of women registered into MM. Data on the number of uploads per month were analyzed using proportions and frequencies. We used confidence intervals at the 95% level to assess statistical differences in rates of active listening across thematic content areas. Data were analysed using Stata 13.2 for five districts: Awutu Senya West, Awutu Senya East, Gomoa West, Ada East and Ada West. The timeline for implementation varied across sites, and spanned from July 2011 to September 2014 overall.

## Results

### Client data application

**Fig. 3:**
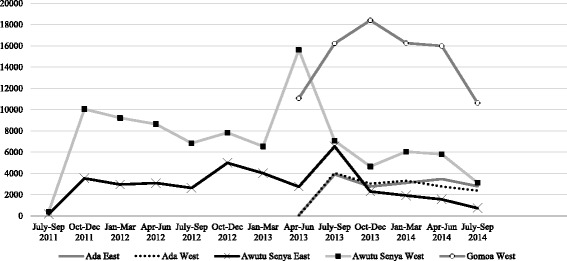
Total data uploads on service utilization by district (Calendar year). Findings highlight variations in data uploads across geographic areas

Figure 3 summarizes mean quarterly uploads on patient level data as part of the Client Data App by district from July 2011 to September 2014. In Awutu Senya East, uploads ranged from 3,534 from July to October 2011 to 2,291 over the same time period in 2013; gradually tampering off as the program drew to a close in September 2014. In Awutu Senya West, the frequency of uploads ranged from 10,044 to 4,647 over the same time period. Trends in Ada West and East were nearly identical at just over 4,000 for July to September 2013 before declining to 3,126 for the same window in 2014. Overall data uploads were highest for Gomoa West ranging from 11,063 in April to June 2013 to 18,403 from October to December 2013, before declining to 10,619 for the period of July to September 2014.

**Fig. 4:**
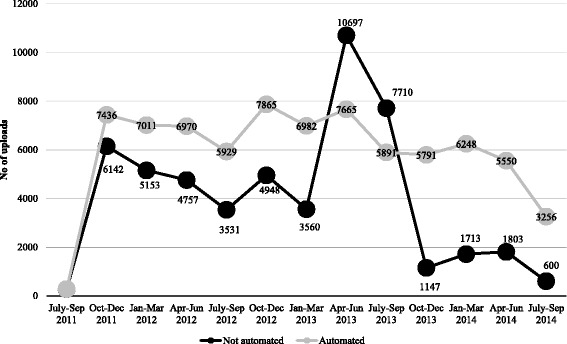
Average data uploads for automated (*n* = 16) vs. non-automated facilities (*n* = 17) from July/Sept 2011–2014 in Awutu Senya East & West

With the exception of Ada West, all CHPS facilities accounted for the over half of data uploads, followed by health centers (Additional file 3: Figure S2). In Gomoa West other health facilities constituted 20% of data uploads, while in Awutu Senya East, a periurban district with a mix of public and private facilities, private clinics accounted for nearly one-quarter of uploads. In Awutu Senya East and Awutu Senya West, automated CHPS facilities (*n* = 16), which receive routine data summaries on trends in data uploading as well as service statistics, were observed to have a greater number of and more stable patterns of data uploading across all facilities and study sites over time (Fig. 4).

### Enrollment into mobile midwife

Table 2 presents data on the characteristics of women enrolled into Mobile Midwife from October 2011 to September 2014 in five districts. A total of 7,370 women were enrolled during pregnancy; 22% during the first trimester, 53% during the second and 25% during the third. Among those enrolled during pregnancy, 85% had a private phone and 71% had had one or more previous children. Across geographic areas, the proportion of women enrolled in the 1^st^ trimester—a factor which has been shown to increase the number of antenatal care visits— was highest in Ada West (37%), followed by Ada East (27%) and Gomoa West (24%). Differences in phone ownership were observed across geographic areas, with greater than 1 in 5 pregnant women in Ada East (22%) and Ada West (27%) sharing phones as compared to 8-19% elsewhere.

**Table 2:** Characteristics of women enrolled into Mobile Midwife from October 2011 to September 2014 in five districts

	Total		Ada East April 2013-Sept 2014		Ada West April 2013-Sep-14		Awutu Senya East July 2011-Sep-14		Awutu Senya West July 2011-Sept 2014		Gomoa West April 2013-Sep-14	
	**n**	**%**	**n**	**%**	**n**	**%**	**n**	**%**	**n**	**%**	**n**	**%**
Pregnant women	**N=**	**7,370**	**N=**	**561**	**N=**	**303**	**N=**	**1,590**	**N=**	**1,323**	**N=**	**3,593**
Gestational age at enrolment	**n=**	**6,607**	**n=**	**459**	**n=**	**266**	**n=**	**1,449**	**n=**	**1,142**	**n=**	**3,291**
1st Trimester	1478	22.4%	125	27.2%	99	37.20%	224	15.5%	226	20.0%	804	24.4%
2nd Trimester	3,498	52.9%	246	53.6%	137	51.50%	830	57.3%	634	55.5%	1651	50.2%
3rd Trimester	1,631	24.7%	88	19.2%	30	11.30%	395	27.3%	282	24.7%	836	25.4%
Phone Ownership	**n=**	**5,932**	**n=**	**501**	**n=**	**259**	**n=**	**1,478**	**n=**	**1,023**	**n=**	**2,671**
Shared	872	14.7%	111	22.2%	71	27.4%	112	7.6%	191	18.7%	387	14.5%
Private	5,060	85.3%	390	77.8%	188	72.6%	1,366	92.4%	832	81.3%	2,284	85.5%
Number of previous children	**n=**	**6,918**	**n=**	**501**	**n=**	**281**	**n=**	**1,506**	**n=**	**1,199**	**n=**	**3,431**
None	1,988	28.7%	142	28.3%	79	28.1%	449	29.8%	357	29.8%	961	28.0%
One	1,680	24.3%	106	21.2%	67	23.8%	390	25.9%	289	24.1%	828	24.1%
2 or more	3,250	47.0%	253	50.5%	135	48.0%	667	44.3%	553	46.1%	1642	47.9%
Postpartum	**N=**	**14,867**	**N=**	**1,454**	**N=**	**1,971**	**N=**	**2,302**	**N=**	**3,693**	**N=**	**5,447**
Age at enrollment	**n=**	**14,843**	**n=**	**1,454**	**n=**	**1,971**	**n=**	**2,300**	**n=**	**3,693**	**n=**	**5,425**
Less than 6 weeks	3,567	24.0%	215	14.8%	460	23.3%	317	13.8%	1134	30.7%	1441	26.6%
7 weeks-6 months postpartum	7,496	50.5%	786	54.1%	962	48.8%	1373	59.7%	1744	47.2%	2,631	48.5%
6-12 months postpartum	3,780	25.5%	453	31.2%	549	27.9%	610	26.5%	815	22.1%	1,353	24.9%
Phone ownership	**n=**	**10,680**	**n=**	**1,259**	**n=**	**1,560**	**n=**	**1,835**	**n=**	**2,111**	**n=**	**3,915**
Shared	2,429	22.7%	327	26.0%	348	22.3%	370	20.2%	577	27.3%	807	20.6%
Private	8,251	77.3%	932	74.0%	1212	77.7%	1,465	79.8%	1,534	72.7%	3,108	79.4%

During the postpartum period, 14,867 women were newly enrolled: 24% within 6 weeks of delivery, 50% between 7 weeks and 6 months postpartum, and 26% between 6 and 12 months postpartum. An estimated 77% of those enrolled postpartum owned a personal mobile phone. Enrollment trends and participant characteristics were similar across geographic areas for postpartum women.

### Message delivery

**Fig. 5:**
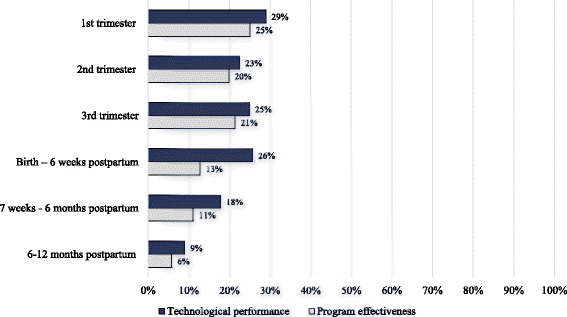
Program effectiveness and technological performance for each stage of the continuum of care from October 2011 to September 30, 2014 in 5 districts of Ghana. The dark blue bars reflect the proportion of messages received out of those expected, while the light grey is the proportion of messages that each woman listened out of the total they were expected to receive including those not received

Figure 5 presents data on the proportion of messages sent out of the total expected for each stage of the continuum of care and by thematic area from October 2011 to September 30, 2014 in 5 districts of Ghana. Across the continuum of care, on average less than one-third of expected messages were pushed out of the system to intended recipients. The largest proportion of messages received occurred in the first trimester (29%). The fewest number of expected messages (9%) were received during the extended postpartum period when an estimated 26 messages were intended to be pushed out from 6 to 12 months following a birth outcome. By thematic area, an average of 12 to 23% of expected messages were received. The fewest number of expected messages received fell under the thematic areas of infant care (12%), postpartum care danger signs (12%), and infant feeding and nutrition (13%).

**Fig. 6:**
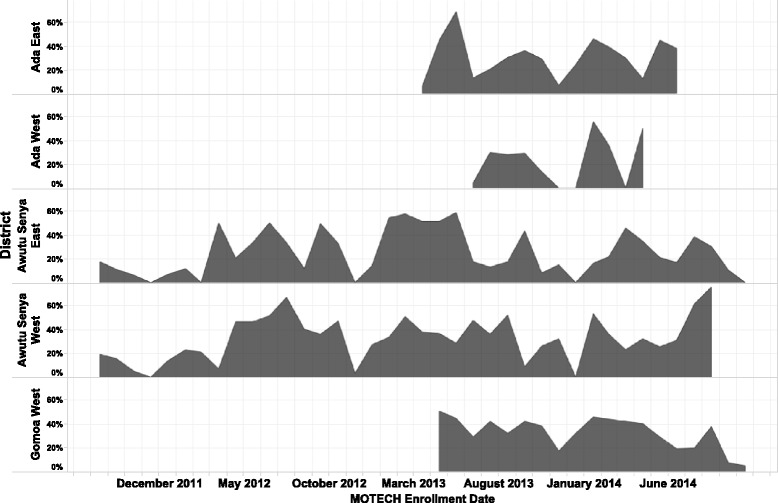
The percentage of messages successfully pushed out to pregnant women from October 2011 to September 2014. The grey area denotes the percentage of messages sent by geographic area over the period of implementation in each site

**Fig. 7:**
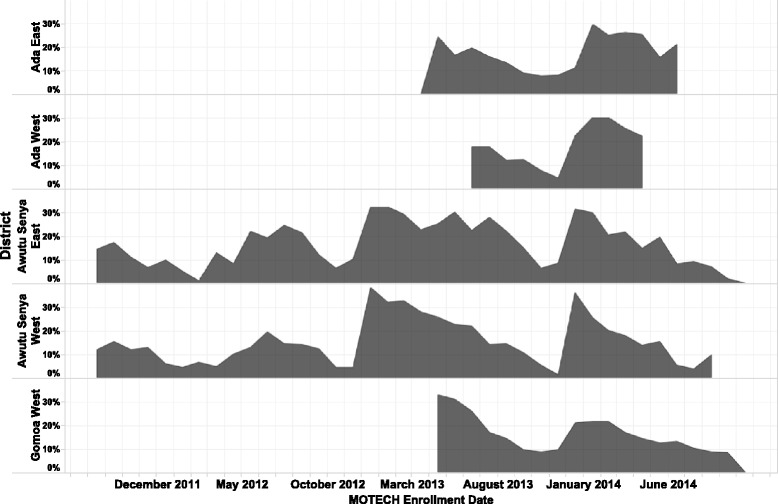
The percentage of messages successfully pushed out to postpartum women from October 2011 to September 2014. The grey area denotes the percentage of messages sent by geographic area over the period of implementation in each site. Message delivery varied across sties and over time, falling under a threshold of 30%

Figures 6 and 7 and Additional file 4: Figure S3 presents data on the percentage of messages successfully pushed out to pregnant and postpartum women, respectively, over time and across geographic areas from October 2011 to September 2014. Message delivery varied across sites and over time, falling under 35% for messages to pregnant women and 20% for messages to postpartum women across all geographic areas (Additional file 4: Figure S3).

### User engagement with messages

**Fig. 8:**
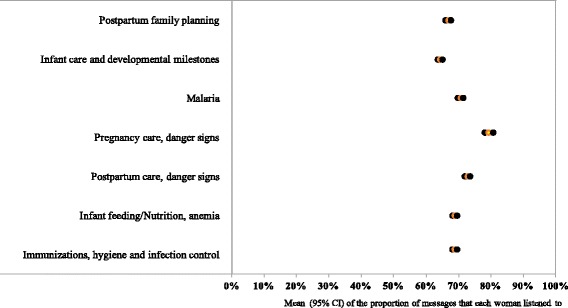
The behavioral performance of Mobile Midwife users assessed by the proportion of messages that each woman listened to out of those received. The yellow dot denotes the mean whilst the black dots reflect the upper and lower bounds of the 95% confidence interval

In addition to data on message delivery, we reviewed the behavioral performance of users by assessing rates of active listening amongst message recipients (Table 3 and Fig. 8). Across the continuum of care from the first trimester to postpartum, 44% to 86% of women who received messages listened to at least 50% the length of each message. Among pregnant women, over 80% chose to listen to messages received; however, postpartum rates of active listening ranged from 44% from birth to post-partum to 54% at 6–12 months postpartum. By thematic area, rates of active listening were significantly higher for messages on malaria (71%), danger signs during pregnancy (79%) and postpartum (73%) than for other content areas (Fig. 8).

**Table 3:** Program effectiveness, technological and behavioral performance

	Program effectiveness (Technological + Behavioral performance)			Technological performance (Platform effectiveness)			Behavioral performance (Messaging exposure)		
	Number of women expected to receive at least one message	Mean of the proportion of messages that each woman listened to overall (including messages not received)	95% CI	Number of women expected to receive at least one message	Mean of the proportion of eligible messages pushed to each woman	95% CI	Number of women who received at least one message	Mean of the proportion of messages that each woman listened to	95% CI
Messages across the continuum of care									
1st trimester	1,618	25.1%	(23.4-26.8%)	1,618	29.0%	(27.5-31.0%)	803	81.0%	(78.5-82.9%)
2nd trimester	3,016	19.9%	(18.8-21.0%)	3,016	22.5%	(21.3-23.6%)	1,266	85.7%	(84.3-87.2%)
3rd trimester	7,242	21.4%	(20.6-22.3%)	7,242	24.9%	(24.0-25.8%)	2,588	81.6%	(80.3-82.8%)
Birth – 6 weeks postpartum	13,763	12.8%	(12.3-13.2%)	13,763	25.7%	(25.1-26.3%)	5,517	43.8%	(42.6-45.0%)
7 weeks - 6 months postpartum	19,688	11.1%	(10.8-11.4%)	19,688	17.8%	(17.5-18.2%)	8,893	53.4%	(52.5-54.2%)
6-12 months postpartum	22,237	5.8%	(5.65-6.0%)	22,237	9.0%	(8.7-9.3%)	6,656	54.2%	(53.3-55.2%)
Messages by thematic areas									
Postpartum family planning	21,216	11.4%	(11.1-11.7%)	21,202	15.9%	(15.6-16.2%)	9,082	66.8%	(66.0-67.6%)
Infant care and developmental milestones	22,237	8.0%	(7.8-8.2%)	22,236	11.5%	(11.3-11.7%)	10,202	64.3%	(63.5-65.0%)
Malaria	20,661	15.0%	(14.7-15.4%)	20,605	20.8%	(20.4-21.2%)	8,540	70.6%	(69.7-71.4%)
Pregnancy care, danger signs	3,735	19.1%	(18.3%-20.0)	3,730	22.9%	(22.0-23.8%)	2,163	79.3%	(78.0-80.6%)
Postpartum care, danger signs	22,214	9.1%	(8.9-9.3%)	22,182	12.1%	(11.9-12.3%)	9,211	72.7%	(71.9-73.4%)
Infant feeding/Nutrition, anemia	22,236	9.6%	(9.4-9.8%)	22,232	13.4%	(13.2-13.7%)	10,909	68.7%	(68.0-69.4%)
Immunizations, hygiene and infection control	22,236	10.2%	(10.0-10.4%)	22,23621	14.2%	(13.9-14.4%)	10,626	68.8%	(68.1-69.5%)

To estimate the overall effectiveness of the program, we estimated overall exposure to messaging content by assessing the proportion of messages that each woman listened to out of the total they were expected to receive including those not received (Table 3). Study findings suggest that exposure to messaging content declined from 25% in the 1^st^ trimester to 6% at 6–12 months postpartum. This decline occurred as the number of eligible MOTECH users increased from 1,618 in the 1^st^ trimester to 22,237 at 6–12 months postpartum. Across thematic areas, the mean proportion of women exposed varied from 19% for pregnancy care danger signs to 8% for infant care and development milestones.

## Discussion

Study findings sought to generate critical insights into the implementation and effectiveness of the MOTECH program—one of the largest, and most comprehensive demand and supply side MNCH mHealth programs ever to be implemented in a low resource setting globally. Program effectiveness was assessed by exploring technological and behavior elements of the program. Technological elements were defined as the MOTECH platform’s ability to ‘push out’ Mobile Midwife messages to pregnant and postpartum women, while behavioral elements included health workers’ willingness to upload patient level data into Client Data App and the willingness of pregnant and postpartum women to listen to Mobile Midwife messages if received (Table 4). The latter, when considered in conjuction with the platform’s ability to deliver messages, provided a measure of overall exposure to critical program content.

**Table 4:** Case study characteristics for investigating the technological and behavioral performance of MOTECH in Ghana

Context	Despite the growing use of technology in the health sector, little evidence is available on the technological performance of mobile health programs nor on the willingness of target users to utilize these technologies as intended (behavioral performance). In this case study of the Mobile Technology for Health (MOTECH) program in Ghana, we assess the platform’s effectiveness in delivering messages, along with user response across sites in five districts from 2011–2014.
Objective	1. Determine what proportion of expected messages are successfully ‘pushed’ out of the MOTECH platform; and 2. Describe differences in rates of active listening among users across study sites, during pregnancy and postpartum, and across thematic content areas.
Study design	Naturalistic
The case	The technological and behavioral performance of the MOTECH program in Ghana
Data collection	System generated data on patient uploads, registration, message delivery and user engagement
Data analysis	Proportions and frequencies; Confidence intervals at the 95% level to assess statistical differences in rates of active listening across thematic content areas.
Key findings	• A total of 7,370 women were enrolled in MM during pregnancy and 14,867 women were enrolled postpartum. • While providers were able to register and upload patient-level health information using CDA, the majority of these uploads occurred in Community-based facilities versus Health Centers where RMNCH client loads are higher. • For MM, 25% or less of expected messages were received by pregnant women, despite the majority (>77%) owning a private mobile phone. • While over 80% of messages received by pregnant women were listened to, postpartum rates of listening declined over time. • Only 25% of pregnant women received and listened to at least 1 first trimester message. • By 6–12 months postpartum, less than 6% of enrolled women were exposed to at least one message.
Limitations	• While data on the number of individuals enrolled into MOTECH are presented, the true denominator from which these individuals are drawn remains unknown. Further details on the characteristics of pregnant and postpartum women not enrolled into Mobile Midwife are also not available. Research at a household level is recommended to better measure the population level coverage and sustained engagement in the program. • Client level analyses of active listening were restricted to unique phone numbers and women with their own phones or access to a phone in their household. Given the common practice of sharing phones and phone numbers, this may have introduced selection bias and led to an overestimate of active listening status. • While we sought to extract data on reminders sent to clients and nurses, these efforts were unsuccessful. Future mHealth platforms should consider ways to improve routine data extraction and use of data to allow for continuous program monitoring. • Finally, efforts to explore linkages between messaging exposure and careseeking were mired by missing data. Future programs should consider ways to improve the accuracy and completeness in data reporting as well as linkages with existing health information systems which collect data on service utilization and reported practices.

MOTECH’s Client Data App was designed to produce automated reminders and alerts, searchable lists of clients needing care, and in two districts (Awutu Senya East and West) where the program had been implemented without encountering prolonged programmatic or political issues, CHPS facilities received automated monthly aggregate reports for upwards reporting. Findings suggest that health care providers were able to upload health information on pregnant and postpartum women enrolled into MOTECH and that the majority of these uploads occurred in frontline CHPS facilities. Variability in the volume and frequency of uploads were observed across all sites, particularly within the first and last quarters of implementation. Facilities that received automated summary reports tended to have more consistent data uploads, however, the usability and reliability of the data in these reports depended heavily on health workers’ mastering clinical data entry and uploading at a high standard of accuracy and consistency. In practice, this proved difficult for the higher-client-volume health centers and hospitals, where overburdened midwives providing much of antenatal, delivery and early postnatal care, and siloed treatment areas meant digitizing patient level data was a low priority. Moreover, the small size of feature phones, specifically their screen and keypad size, likely contributed to inability to community health nurses and other staff to maintain consistent data uploads. Because of these challenges, none of the district health centers achieved the 85% accuracy and completeness level required to allow meaningful use of automated reports [13]. In contrast, at the community level, health facilities were better able to attain accuracy and completeness level targets as demonstrated in Awutu Senya, where all the facilities achieved the accuracy threshold and nurses even went on to train others in the system. Overall, a mobile-based client data system like MOTECH’s Client Data App can be of real value, particularly at the community-level among health workers that are manageable patient volumes. At the facility-level, maintaining data accuracy and completeness is likely to hinge upon added human resources/support given that certain cadres of health workers are currently overburdened with patient volumes, as well as larger mobile devices for data entry. Strong district leadership, consistent monitoring of data quality, and reliable network access were all essential optimizing the use of Client Data App [13].

Mobile Midwife sought to improve knowledge, awareness, and utilization of MNCH services through exposure to health information messages, and care reminders and alerts. In contrast to other mHealth programs which have used SMS delivery channels [16] to push out information messages, the majority of MOTECH participants opted to receive information in local languages through voice delivery channels. Delivery through this modality may improve access and awareness among women with limited to no literacy—an estimated 30% of all Ghanians. Despite this potential, and message delivery system’s ability to be persistent when calls are listened to for less than 30 s, quarterly fluctuations were observed in the proportion of expected messages pushed out of the system and overall, only two-thirds of expected messages were delivered. The precise factors underpinning system failures in message delivery are unknown due to the way the system logs the call failures [13]. However, they are likely due to one or more of the following reasons: inconsistent network connectivity, call congestion in the E1 phone lines as more clients were enrolled in the program, client phones’ being powered off because they were not charged or unable to be charged because of inconsistent access to the power grid, and, finally, technical problems within the MOTECH system [13].

Message delivery challenges occurred despite extensive efforts to conduct performance testing and upgrade the software and devices used. In 2012, Grameen Foundation significantly revised the open-source MOTECH software platform to expand the range of features and make it more robust for larger-scale deployments by multiple organizations internationally. At this time, performance/stress testing was conducted by sampling 20,000 subscribers and sending messages out to them all at the same time. While findings from the performance testing suggested that the system performed well and was capable of sending 120 messages per minute for SMS and data communications, voice calls were identified as a bottleneck. In particular, the program was constrained by the availability of E1 phone lines to deliver voice messages. Services drew upon two active lines available in Ghana, which together supported 60 simultaneous calls. As a way to accommodate a growing number of clients enrolling into the program, outbound calls were distributed more evenly over the course of a day, and eventually a “queue” system was introduced in the software to ensure that the maximum number of simultaneous calls were not exceeded. Grameen Foundation also housed a customer support center with staff capable to dealing with low tech issues in both Client Data App and Mobile Midwife, and a streamlined process of moving complicated software issues to the tech/IT department. In the first half of the program period, staff received inquires from clients and health workers not just on registration, data uploads, and message delivery, but also on MNCH-related care, something Grameen Foundation staff were not trained to handle. As a result of this, as well as commitment to build district-level capacity to manage Mobile Midwife, responsibilities of client feedback and inquiries were transferred to the DHMTs, and the customer support center only dealt with technological issues with Mobile Midwife raised by the districts. Given that nearly all MOTECH enrollees opted to receive automated voice over SMS messages—a factor which when considered with the number of available phone lines and passive monitoring of message delivery, may explain MOTECH-system related delivery challenges. Future programs should more carefully weigh the options on messaging format given to clients with the feasibility of message delivery based on consideration of the number of available phone lines, message size, and number. Systems for the continuous extraction and assessment of data and simple dashboards tracking message delivery should additionally be instituted from the program’s inception to identify trends in the proportion of expected messages received over time by delivery channels (SMS, voice, USSD), and content area in real-time. Unfortunately, our evaluation occurred retrospectively in 2015 and not in time enough to yield meaningful changes in the MOTECH program or platform.

Beyond the MOTECH platform’s technical performance in message delivery, we sought to derive a measure of overall ‘exposure’ by determining the proportion of women who actively listened to the messages received. To measure the dose of exposure, we defined ‘active listening’ as the proportion of women who listened to at least 50% of content of 50% of messages. While we explored alternative thresholds, we opted for the 50% threshold based on the assumption that individuals would have the capacity to glean the bulk of topical content in that window of time. Future analyses may wish to assume more stringent thresholds of 100% and additionally interview clients to identify whether messages were shared with family members and/or listened to multiple times. Findings on active listening suggested that overall willingness to listen to messages exceeded 60% for all topical areas. However, rates of active listening were observed to decline during the postpartum window which may suggest that there is a ‘novelty’ effect associated with mobile messaging and/or that alternative content formats are needed to rekindle interest (e.g. quizzes, narrative stories, etc.). Future programs may also need to consider an abbreviated number of messages better targeted to address critical gaps in practices and/or careseeking.

To explore the effectiveness of exposure to messaging we initially sought to evaluate the careseeking patterns of women enrolled into Mobile Midwife, including the timing and utilization of services across the continuum of care. However, these data have not been presented because a large proportion of data were missing, and/or unable to be linked to the data on listening. This is part due to the fact that clients move between public and private facilies for MNCH care, and the difficulty of maintaining data uploads in high patient volume facilities, such as health centers and hospitals, where much of that critical care is provided. Future efforts to evaluate the effectiveness of health information messages in bolstering careseeking, should address this critical evidence gap by assessing the effects of specific bundles of messaging based on enrollment during pregnancy or postpartum, by thematic area and across the continuum of care.

This is first analysis of its kind to examine large volumes of back-end data to draw conclusions about the effectiveness of a large-scale mHealth program. Study findings aim to encourage transparency in presenting data on digital health platforms and foster wider dialogue on how to optimize these systems to improve the accessibility and use of data to inform implementation and evidence on their effectiveness. The increased availability of data as part of mHealth programs offers much promise for improving and measuring the exposure of target users to critical program content and allowing for the routine use of patient-level health information at multiple levels of the health system. Study findings highlight importance of real-time program monitoring to protect against ‘program drift’ and ensure the functionality of the underlying technical platform as well as the continued engagement of target users. Efforts to scale-up and sustain the use of mobile phones and other digital devices to improve service delivery in health facilities will need to ensure the adequacy of human resources along with strong district leadership, consistent monitoring of data quality, and reliable network access. Challenges in the delivery of health information messages to pregnant and postpartum women limited the program’s reach and exposure. While over 80% of messages received by pregnant women were listened to, declines in postpartum rates of active listening highlight the need for further research to identify the factors underpinning user engagement over time.

### Comparison with other studies

Calls to improve the rigor of mHealth evidence generation and reporting are emerging [17]. Findings from a systematic review of mHealth solutions on coverage and use of ANC, PNC, and childhood immunizations in low- and middle-income countries suggests that there is some evidence of effectiveness at changing behavior [18]. Among specific studies, two RCTs—one in Zanzibar and one in Kenya— which explored the effectiveness of text message reminders and education delivered to pregnant women’s mobile phones, found evidence of statistically significant increases in ANC in their intervention groups relative to their control groups [16, 19]. In Zanzibar, program activities represent one deployment of the Mobile Alliance for Maternal Action (MAMA) project which has additional sites in Bangladesh, Malawi, India, and South Africa and like MOTECH aims to improve MNCH outcomes through the delivery of timed health information messages and appointment reminders. While evaluation activities are still underway for many MAMA deployments, published findings from Zanzibar have emphasized summative findings only; suggesting not only increases in the proportion of women receiving the recommended four ANC visits during pregnancy as well as a trend towards improved quality of care^4^ [16]. In Kenya, a similar initiative of health information messages and service prompts corresponded to an increase in adherence to recommended ANC visits [19]. In both settings, the absence of details on the technological platforms which serve as the backbone of these programs greatly limits understanding of the factors influencing effects observed, their comparability with alternative resource uses, and generalizability to other settings. While the word count limitations of peer review manuscripts may hinder efforts to report the details of *all* evaluation activities, robust evaluations should consider the technological performance of the program as a key component of its impact pathway and in particular, its ability to perform as intended. As findings from MOTECH demonstrate, the technological performance of a program is subject to change over time and as scale increases. The failure to monitor this may impact exposure to the program and in turn findings on its effectiveness.

Elsewhere in the literature, a similar pattern of mHealth program evaluations providing limited details on the technological performance of mHealth programs is emerging. Evaluation findings of Text2Floss—a 7-day text messaging intervention which sought to improve oral health behavior and knowledge in the US— suggest that the program led to improvements in knowledge and oral health outcomes [20]. While the characteristics of the automated, multichannel, two-way SMS gateway text messaging service are described, evaluation findings do not provide details on the performance of the technology platform, including messages sent and received out of those expected. Elsewhere in the US, a report on the lessons learnt from a randomized controlled trial of an mHealth smoking cessation program does define the technology functionality by the “pathing of participants from one stage to the next” and report recording these data by the software program, however, no additional data or details are presented [21]. Similarly in Australia, findings from a process evaluation of the TXT2BFIT mHealth program designed to improve weight management in young adults (18–35 years) presented details on the number of text messages delivered and replied to from MessageMedia® web platform; suggesting high rates (98%) of message delivery [22]. However, broader details on the platform were not well detailed [22]. Beyond summative evaluations, qualitative studies have sought to explore user perceptions of mobile tools and/or health information messaging programs in the US [23, 24], New Zealand [25], and India [26–29]. However, none of these present quantitiative data on the technological performance of the program; instead focusing on reported perceptions of the technology, including feasibility, usability and/or acceptability.

### Limitations

There are several limitations to our analyses (Table 4). Implementation of the MOTECH program relies on an initial period of client registration by health workers. While data on the number of individuals enrolled into MOTECH are presented, the true denominator from which these individuals are drawn remains unknown. Similarly, study findings are limited by the absence of details on pregnant and postpartum women not enrolled into Mobile Midwife. Research at a household level is recommended to better measure the population level coverage and sustained engagement in the program. Client level analyses of active listening were restricted to unique phone numbers and women with their own phones or access to a phone in their household. Given the common practice of sharing phones and phone numbers, this may have introduced selection bias and led to an overestimate of active listening status. Health worker use of the Client Data App is limited in this analysis to absolute numbers on the numbers of patients seeking care uploaded into the system. Details on the frequency and timing of these uploads coupled with the completeness of records is missing. Given the challenges reported with the Mobile Midwife message delivery platforms, it is feasible that similar challenges were present in the uploading of patient level information by health workers.

Future research is recommended to more closely track the flow of data across recipients and levels of the health system over time, particularly within and across districts. While we sought to extract data on reminders sent to clients and nurses, these efforts were unsuccessful. Future mHealth platforms should consider ways to improve routine data extraction and use of data to allow for continuous program monitoring. Finally, with regard to message delivery platforms, considerable challenges were noted in the preceding sections. Beyond these, added details on the length of each message, in addition to the number and format, are needed to formulate recommendations on the optimal data package appropriate for use in a program. Since messaging programs can be costly, it is further recommended that linkages between messaging exposure and careseeking be assessed and the value for money of this component weighed against alternative resource uses.

## Conclusions

Digital health programs should not assume that messaging programs perform as intended both with regard to technical functionality and the behavioral responses of end-users. Evaluations of mobile health programs should measure technological, behavioral, health systems, and/or community factors which may lead to breaks in the impact pathway and ultimately, influence findings on program delivery and effectiveness.

## Additional files

Additional file 1: Figure S1. Data flows through the MOTECH system reproduced with permission from [1]. (DOCX 150 kb)
Additional file 2: Table S1. Summary metrics for assessing program effectiveness including technological and behavioral components. (DOCX 31 kb)
Additional file 3: Figure S2. Proportion of data uploads by facility type and district. Trends in system generated data on uploads by facility type and district. (DOCX 133 kb)
Additional file 4: Figure S3. Proportion of messages sent out of those expected across the continuum of care by district. Trends in system generated data on message delivery. (DOCX 73 kb)

## Abbreviation

ANC: Antenatal care
CHN: Community health nurse
CHPS: Community-based health planning and services
GHS: Ghana health services
MAMA: Mobile alliance for maternal action
mHealth: Mobile health
MNCH: Maternal, newborn, and child health
MOTECH: Mobile technology for community health
SMS: Short messaging service

## Footnotes

1 Early deployments in 2010 relied on a Nokia 1680c-2 feature phone. These were upgraded to the Nokia 2330c-2 and Nokia C1-01, and later to the Nokia Asha 200 to enhance functionality and usability through features such as dual SIM capacity and a larger screen and full keyboard that made it easier to enter data and reduced entry errors. To replace aging Nokia 2330c-2 s and to test a smartphone (Android) version of the Client Data AppClient Data AppClient Data AppClient Data AppClient Data Application, Samsung Galaxy S4 phones were used in the Upper East region.

2 Facility-level health workers include Community Health Nurses, Midwives, data entry staff, and health extension workers working at CHPS, Health Centers and Hospitals

3 Initially, this service also provided support for clients enrolled in Mobile Midwife, however, midway through the program, monitoring and feedback from clients were transferred to the districts as part of program capacity building.

4 Measured by increasing the identification and referral of antepartum complications, increases in the number of women receiving preventive health services and attending ANC late in pregnancy.
