# Supplementary material for: Mobile Technology for Community Health in Ghana: what happens when technical functionality threatens the effectiveness of digital health programs?
Source: BMC Med Inform Decis Mak. 2017 Mar 14;17:27. doi: 10.1186/s12911-017-0421-9 (PMC5351254; doi:10.1186/s12911-017-0421-9)
Supplement: Additional file 1: Figure S1. — Data flows through the MOTECH system reproduced with permission from [1]. (DOCX 150 kb) [file 12911_2017_421_MOESM1_ESM.docx]

**Supplementary Web Figure 1. Data flows through the MOTECH system reproduced with permission from [1]**

**
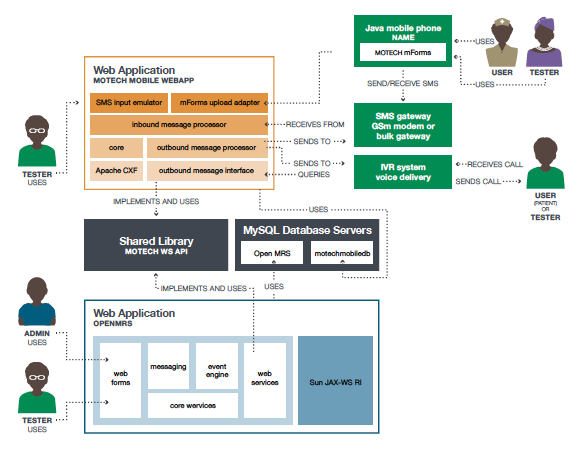
**

1. Foundation, G., *Using mobile technology to strengthen maternal, newborn, and child health: a case study of MOTECH's five years in rural Ghana*. 2015, Grameen Foundation: Washington, DC.
