# Supplementary material for: Mobile Technology for Community Health in Ghana: what happens when technical functionality threatens the effectiveness of digital health programs?
Source: BMC Med Inform Decis Mak. 2017 Mar 14;17:27. doi: 10.1186/s12911-017-0421-9 (PMC5351254; doi:10.1186/s12911-017-0421-9)
Supplement: Additional file 2: Table S1. — Summary metrics for assessing program effectiveness including technological and behavioral components. (DOCX 31 kb) [file 12911_2017_421_MOESM2_ESM.docx]

**Supplementary Web Table 1. Summary metrics for assessing program effectiveness including technological and behavioral components**

| **Domain** | **Indicator** | **Numerator** | **Denominator** |
| --- | --- | --- | --- |
| Technological component | Proportion of messages pushed out of those expected | Number of messages pushed as recorded by message logs | Total number of messages expected to be received |
| Behavioral component | Proportion of messages that each woman listened to out of the ones received | Number of messages listened for at least 50% of the length of each message | Total number of messages pushed to eligible women |
| Overall Program effectiveness | Proportion of messages that each woman listened to out of the total they were expected to receive (including those not received) | Number of messages listened for at least 50% of the length of each message | Total number of messages expected to be received |
