# Supplementary material for: Mobile Technology for Community Health in Ghana: what happens when technical functionality threatens the effectiveness of digital health programs?
Source: BMC Med Inform Decis Mak. 2017 Mar 14;17:27. doi: 10.1186/s12911-017-0421-9 (PMC5351254; doi:10.1186/s12911-017-0421-9)
Supplement: Additional file 3: Figure S2. — Proportion of data uploads by facility type and district. Trends in system generated data on uploads by facility type and district. (DOCX 133 kb) [file 12911_2017_421_MOESM3_ESM.docx]

**Supplementary Web Figure 2. Proportion of data uploads by facility type and district**
