# Supplementary material for: Mobile Technology for Community Health in Ghana: what happens when technical functionality threatens the effectiveness of digital health programs?
Source: BMC Med Inform Decis Mak. 2017 Mar 14;17:27. doi: 10.1186/s12911-017-0421-9 (PMC5351254; doi:10.1186/s12911-017-0421-9)
Supplement: Additional file 4: Figure S3. — Proportion of messages sent out of those expected across the continuum of care by district. Trends in system generated data on message delivery. (DOCX 73 kb) [file 12911_2017_421_MOESM4_ESM.docx]

**Supplementary Web Figure 3. Proportion of messages sent out of the total expected for each stage of the continuum of care from October 2011 to September 30, 2014 in 5 districts of Ghana**. The n represents the total number of expected messages sent to women enrolled.
